# Pharmacist medication review: An integrated team approach to serve home-based primary care patients

**DOI:** 10.1371/journal.pone.0252151

**Published:** 2021-05-25

**Authors:** Michele Monzón-Kenneke, Paul Chiang, Nengliang (Aaron) Yao, Mark Greg

**Affiliations:** 1 Northwestern Medicine, Evanston, IL, United States of America; 2 Home Centered Care Institute, Schaumburg, IL, United States of America; 3 Center For Health Management and Policy, School of Public Health, Cheeloo College of Medicine, Shandong University, Jinan, Shandong, China; 4 Section of Geriatrics, University of Virginia, Charlottesville, VA, United States of America; Nord University, NORWAY

## Abstract

**Background:**

Comprehensive medication review is a patient-centered approach to optimize medication use and improve patient outcomes. This study outlines a pilot model of care in which a remote corporate-based clinical pharmacist implemented comprehensive medication reviews for a cohort of medically complex home-based primary care (HBPC) patients.

**Method:**

Ninety-six medically complex patients were assessed for medication-related problems. Data collected on these patients were: number of chronic conditions, number of medications, appropriate indication for each medication, dose appropriateness, drug interactions, recommendations for medication optimization and deprescribing. The number of accepted recommendations by the HBPC practice was analyzed.

**Results:**

On average, the patients were 82 years old and had 13 chronic conditions. They were taking a median of 17 medications. Over a four-month pilot period, 175 medication recommendations were made, and 53 (30.3%) of them were accepted, with most common being medication discontinuation, deprescribing, and dose adjustments. Sixty-four (66.7%) patients were on a medication listed as potentially inappropriate for use in older adults. The most common potentially inappropriate medication was a proton-pump inhibitor (38.5%), followed by aspirin (24%), tramadol (15.6%), a benzodiazepine (13.5%) or an opioid (8.3%). Eighty-one medications were recommended for deprescribing and 27 medications were discontinued (33.3%). There were 24 recommended dose adjustments and 11 medications were dose adjusted (45.8%). Thirty-four medications were suggested as an addition to the current patient regimen, 2 medications were added (5.9%).

**Conclusion:**

Pharmacist comprehensive medication review is a necessary component of the HBPC healthcare continuum. Additional research is needed to examine whether aligning pharmacists to deliver support to HBPC improves clinical outcomes, reduces healthcare expenditures and improves the patient’s experience.

## Introduction

Clinical pharmacists play an essential role within interdisciplinary teams in optimizing medication use, alerting providers to gaps in care, decreasing inappropriate prescribing practices and improving medication safety [1, 2]. Comprehensive medication review is a patient-centered approach to optimize medication use and improve patient outcomes by ensuring each patient’s medication is assessed for indication, effectiveness and safety given patient status and comorbidities [3]. Physicians in ambulatory settings often have limited access to a dedicated pharmacist resource [1–3]. This study sought to implement a remote corporate-based pharmacist into a home-based primary care practice to facilitate comprehensive medication reviews.

About 2 to 4 million Americans have difficulty obtaining office-based primary care because they are frail, functionally limited, chronically-ill and/or homebound [4, 5]. These “invisible” people are the most expensive patients [6], and they fall through the cracks of our current healthcare delivery system. When in need, they often turn to emergency services for medical help but have no continuous, follow-up care [7]. This continues a cycle of poor health management and high expenses. This population is expected to grow dramatically as our society continues to age. The home-based primary care (HBPC) model offers an opportunity to meet their demand and save healthcare costs [5]. HBPC brings the expertise of primary care providers and the technology of a health care clinic directly to medically complex patients, providing comprehensive, coordinated care in the comfort of their home.

Limited knowledge exists on the integration of pharmacist support in private sector HBPC practice [8]. Clinical pharmacist’s role is widely known within outpatient retail settings and hospital inpatient interdisciplinary teams. However, clinical pharmacy support of ambulatory based medical practices is limited [9], including in HBPC [10]. As the population ages and the option of many medical services being made available from home, it is important to include all of the services available to patients who standardly seek care in traditional settings. A remote-pharmacist functions as a liaison transcending novel healthcare landscapes providing oversight essential for safe medication use. Comprehensive medication management is a critical function that assists in improving medication use, especially in those utilizing many medications to manage their multiple coexisting disease states. Older adults using multiple medications may be at risk of medication-related problems leading to adverse health outcomes [11]. Comprehensive medication management in the HBPC population is crucial as these patients have multiple comorbidities and most fit the criteria for polypharmacy. Polypharmacy’s definition can be variable but is commonly considered to be the use of five or more medications [12]. Polypharmacy has been associated with increased risks of adverse events and poor health outcomes [12]. Polypharmacy can also lead to countless medication-related problems (MRP).

Literature searches for remote-pharmacist medication management in home-based primary care did not yield any studies. The demographic of the HBPC patient in this pilot is characterized as having multiple comorbidities, elderly and overwhelmed by polypharmacy. Globally, medication safety in older adults impacts health outcomes and is an enduring health issue. Medication-related problems can be the cause of hospital admissions and cause significant morbidity and mortality. Thirty percent of hospital admissions may be a result of an adverse drug reaction, of which 53.4% are considered preventable [13]. Adverse drug reactions cause significant morbidity and mortality especially as patients age, with a patient aged 75 years or older at the greatest risk [14].

This pilot population of HCBP was compromised of persons of advanced aged and medically vulnerable. Utilizing a remote-pharmacist service can support a HBCP practice by assisting in illuminating medication-related problems. In a study by Vink et al. pharmacists were able to identify medication-related problems in home care patients that were not identified from other providers [15]. In that study the most common problems identified were suboptimal therapy and using of unnecessary medications [15]. A review article describing medication-related problems in home care, commonly noted MRPs were due to potentially inappropriate medications, medication errors and adverse drug reactions [16]. In that same study, it was relayed that teams lacking an interdisciplinary model had patients who were at risk for experiencing MRPs [16].

The Northwestern Medicine Physician Network (NMPN) Accountable Care Organization (ACO) includes over 3,100 providers and approximately 400,000 covered lives. Many of these providers, including HBPC physicians and nurse practitioners, have expressed the need for pharmacist resources to assist with general drug information and patient specific medication consult support. This pilot study was a collaboration with Northwestern Medicine (NM) Regional Medical Group (RMG) Home Care Physicians who provide primary care to medically complex patients in their homes. The majority of these patients are older adults who live alone and have functional disabilities making it difficult to travel or leave their homes to obtain medical care. As strong proponents of team-based healthcare, Home Care Physicians requested assistance from the NMPN Pharmacy Team to review and offer feedback on their patient’s medication regimens. The aim of this study was to describe this pilot program and examine the degree of medication recommendation acceptance by the HBPC practice.

## Methods

### Pilot innovation

This program was created to assist our pilot providers with medication management. Our ACO members expressed the need for pharmacist intervention in assisting with their patient care. Many of our members have no dedicated pharmacy resource and have stated that inpatient and outpatient pharmacists do not have the time or access to the patient medical records to provide comprehensive medication reviews. This program is innovative because this is the first program to incorporate a remote-pharmacist into a HBPC practice. There are no studies that have examined this type of team structure.

No clinical pharmacist service existed at the practice prior to the intervention. The pharmacist performing the medication reviews was employed by the ACO. The pharmacist performing the reviews has two board certifications: A Board-Certified Pharmacotherapy Specialist and a Board-Certified Geriatric Pharmacist with extensive experience in the ambulatory care setting managing patient with complex conditions.

The NM RMG Home Care Physician Team includes 2 physicians and 3 advanced practice nurses that serve approximately 750 patients.

### Medication review

Over a four-month pilot period, a total of 96 patient charts were reviewed by one clinical pharmacist. The average time spent on each patient’s chart review and medication history was approximately 45 minutes. The total time allotted to the project was about 100 hours or 1 hour per patient. This time included chart review, literature review and guideline research in support of recommendations, messaging providers, and recording interventions in a Microsoft Excel spreadsheet.

The program workflow emanated from a weekly email received by the pharmacist containing a list of new Home Care patients on 5 or more medications to review for that week. Patient name, medical record number, and date of birth were forwarded to the pharmacist. The pharmacist would research the patient in the electronic medical record (EMR). Review consisted of reading patient notes, history and physical, laboratory (lab) results, and the medication list. Initially, the pharmacist would review all the patient’s medications and medical history and ensure that each medication prescribed for that patient was appropriate. Medication reviews were performed in a systematic manner by a single pharmacist. As part of a pharmacist’s training, they perform prospective reviews and determine indications for medication use, correct dosage and directions, duplication of therapy, medication effective for condition (based on current patient status and lab results), symptom management recommendations, and patient-centered considerations (affordability, alternative formulations).

A Microsoft Excel spreadsheet was created and consensus regarding outcomes of interest was agreed upon by the pilot team. The spreadsheet contained several headings allowing for methodical review of each patient capturing demographics; provider name; number of chronic conditions; number of medications; renal/hepatic dosing appropriate; patient currently on a American Geriatric Society Beers List medication; name of American Geriatric Society Beers List medication; drug-drug interaction; number of medications recommended for deprescribing; name of medication recommended for deprescribing; additional medication recommended; name of medication recommended; number of total recommendations; number of recommendations taken and intervention taken by provider.

Determination of the number of chronic diseases a patient had was achieved by reviewing the Problem List in the EMR. Chronic medical conditions were defined mirroring the definition as described by the Centers for Disease Control and Prevention (CDC), a condition lasting one or more years that requires ongoing medical attention and/or limits the activities of daily living.

All medications that were current on the patient medication list in the EMR were totaled and considered the patient’s total medication count. This included regularly scheduled medications, as needed medications and medications taken during a specific time period such as an antibiotic.

The completed Excel spreadsheet was sent back to the providers each week. In addition to the spreadsheet, individual messages were sent to providers alerting them of patients in which the pharmacist recommended adjustments for hepatic/renal dosing, significant drug-interactions, and changes in medications based on laboratory results. Additionally, patients were also brought to the provider’s attention if they had a chronic condition that could benefit from dose titration or augmentation of therapy. PubMed and disease specific guidelines were used in assisting with recommendations. Information for recommendations to augment current treatment was gleaned from the pharmacist’s knowledge and experience in ambulatory care. Knowledge was supported by practice guidelines for major chronic illnesses. For example, for diabetes (American Diabetes Association–ADA Standards of Medical Care in Diabetes), chronic obstructive pulmonary disease (Global Initiative for Chronic Obstructive Lung Disease—GOLD Guidelines), hyperlipidemia (American College of Cardiology/American Heart Association Cholesterol Practice Guidelines), chronic kidney disease (Kidney Disease Improving Global Outcomes–KIDIGO), depression (American Psychiatric Association).

Drug interactions were reviewed using Micromedex. Dose adjustments were based on estimated glomerular filtration rate (eGFR) or Cockcroft-Gault Equation for creatinine clearance (CrCl) as suggested by the medication prescribing information.

### Measures

Patients were assessed for number of chronic conditions, number of active medications prescribed, appropriate indication for each medication, dose appropriate for renal, hepatic, age or other specific monitoring parameters, medication listed on the American Geriatric Society Beers Criteria, drug-drug interactions, medications to consider for deprescribing, medications to add to current therapy to optimize disease state treatment.

At the end of the pilot program, the charts of those patients were re-reviewed to determine how many of the recommendations provided were accepted.

### Analysis and approach

We first performed a frequency analysis of the baseline characteristics of the HBPC patients that received pharmacist medication review. At the end of the pilot period, a cross-table was created to show the number of recommendations per patient and accepted recommendations. A frequency table of the potentially inappropriate medications was included in the analysis. We then selected three patients to describe a detailed report of their medication deprescribing and optimization.

Summary of the pilot results was shared with the providers at NM RMG Home Care. The senior medical advisor (physician champion) reviewed findings with his staff to better understand the reasons why many of the recommendations from the pharmacist were not accepted. The senior advisor interviewed the providers to understand the common barriers in implementing the recommendations. They also discussed the role a pharmacist has to assist with medication optimization and deprescribing. We have summarized their discussions in the results section.

## Results

The average age of the pilot population was 82 years. Table 1 shows that over a third of the patients were 85 years or older. About 61% are female. About 71% of them were enrolled in the traditional Medicare program. On average, the patients had 13 chronic conditions and were taking a median of 17 medications. About 70% of these patients were taking 15 or more medications (Table 1).

**Table 1:** Baseline characteristics of the home-based primary care patients received pharmacist medication review (N = 96).

	N	Std
Age, N (%)		
Younger than 65	22	22.9%
65–74	17	17.7%
75–84	23	24.0%
85 or older	34	35.4%
Sex, N (%)		
Male	37	38.5%
Female	59	61.5%
Payers, N (%)		
Traditional Medicare	68	70.8%
Medicare Advantage	20	20.8%
Medicaid	8	8.3%
Chronic Conditions, N (%)		
5–8	23	24.0%
9–12	41	42.7%
13–32	32	33.3%
Medications, N (%)		
8–14	29	30.2%
15–19	35	36.5%
20–47	32	33.3%

The clinical pharmacist made 175 recommendations, and 53 (30%) were accepted by the HBPC providers. While about 19% of patients did not receive any recommendations, about 28% and 29% of patients have received one and two recommendations, respectively (Table 2). Among 78 patients receiving recommendations, HBPC providers accepted at least one recommendation for 40% of these patients.

**Table 2:** Recommendations from pharmacist medication review and acceptance by home-based primary care providers.

	Number of Accepted Recommendations, N (Row %)					Row Total (Column %)
	Zero	One	Two	Four	Seven	
**Number of Recommendations**						
Zero	18 (100%)					**18 (18.8%)**
One	17 (63.0%)	10 (37.0%)				**27 (28.1%)**
Two	18 (64.3%)	5 (17.9%)	5 (17.9%)			**28 (29.2%)**
Three	7 (58.3%)	3 (25.0%)	2 (16.7%)			**12 (12.5%)**
Four	3 (50.0%)	2 (33.3%)		1 (16.7%)		**6 (6.3%)**
Five	2 (100%)					**2 (2.1%)**
Seven		1 (50%)			1 (50%)	**2 (2.1%)**
Eight					1 (100%)	**1 (1.0%)**
**Column Total (Row %)**	**65 (67.7%)**	**21 (21.9%)**	**7 (7.3%)**	**1 (1.0%)**	**2 (2.1%)**	**96 (100%)**

The most commonly heeded intervention was medication discontinuance or deprescribing and dose adjustments. Eighty-one medications were recommended for deprescribing and 27 medications were discontinued (33%). There were 24 recommended dose adjustments and 11 medications were dose adjusted (46%). Eleven medications were suggested as an addition to the current patient regimen.

**Fig 1:**
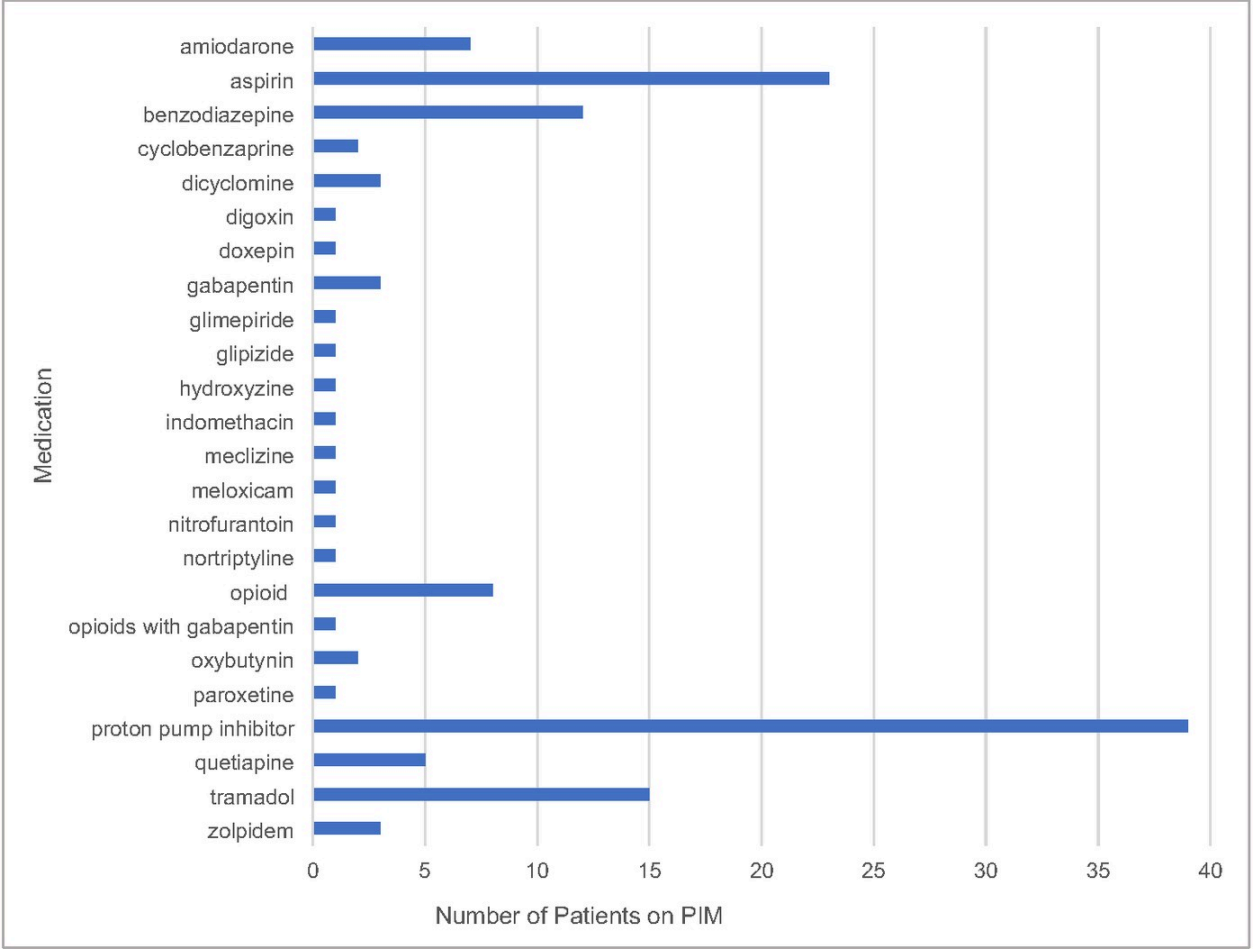
Frequency of potentially inappropriate medications in home care patients (N = 96).

Sixty-four (67%) of the 96 patients were on medication listed as potentially inappropriate on the American Geriatric Society Beers Criteria, 11 patients were not on a Beers List medication and in 21 patients the criteria were not applicable given current age. Fig 1 shows the most common potentially inappropriate medication was a proton-pump inhibitor (41%), followed by aspirin (24%), tramadol (16%), a benzodiazepine (14%) and an opioid (8%).

Pharmacist intervention in HBPC improved patient safety and had financial implications. Table 3 describes three examples of pharmacist recommendations that were accepted. In the first example, a 79-year-old patient on eltrombopag required medication dose adjustment based on their lab results to prevent potential thromboembolism; the second accepted recommendation was for medication consolidation and tapers in a 49-year-old patient taking several central nervous system (CNS) depressants who was at high risk for adverse drug reactions due to concomitant cannabis use and multiple comorbidities. In the third example, a 93-year-old patient on concomitant warfarin and torsemide was switched to apixaban to avert a drug interaction which prior to the modification was the cause of months of not achieving the international normalized ratio (INR) goal. Financial implications are added to each recommendation to emphasize how these interventions could have resulted in health care cost avoidance.

**Table 3:** Three case studies of accepted medication recommendations.

**79-year-old patient on eltrombopag for idiopathic thrombocytopenia (ITP)**
• Labs—platelets– 451 x 10 (3) uL
• Per Micromedex drug information on eltrombopag [17], dose adjustment required for platelet counts above 400 x 10(9)/L, in ITP.
• Provider messaged potential dose adjustment required.
**Safety:** Thromboembolism (venous or arterial) may occur with excessive increases in platelet levels. Incidence of thrombosis in ITP– 6%.
**Financial Implications:**
“Treatment of an acute VTE on average appears to be associated with incremental direct medical costs of $12,000 to $15,000 (2014 US dollars) among first-year survivors, controlling for risk factors. Subsequent complications are conservatively estimated to increase cumulative costs to $18,000–23,000 per incident case. Annual incident VTE events conservatively cost the US healthcare system $7–10 billion each year for 375,000 to 425,000 newly diagnosed, medically treated incident VTE cases [18].”
**49-year-old patient on multiple CNS depressants, opioids, benzodiazepines, SSRI and z-drug at high doses**
• Recommendation to consider medication tapers and consolidation of therapy.
• Patient mentions to provider they had previously used cannabis. Provider tests patient and they are positive for cannabis.
• Note to provider that components in marijuana can interfere with CYP450 enzymes competitively inhibiting the metabolism of other compounds [19]. This interaction could impact benzodiazepines, opioids and CYP2D6 which metabolizes SSRIs and could potentially explain need for increased doses.
**Safety:** With the legalization of marijuana in many states, it is imperative for providers to question patients regarding the use of cannabis products. Medically complex patients with multiple comorbidities are at risk for adverse drug reactions.
**Financial Implications:**
“The average direct costs per patient caused by ADEs were USD $444.90 [95% CI: 264.4 to 625.3], corresponding to USD $21 million per 100,000 adult inhabitants per year. Inpatient care accounted for 53.9% of all direct costs caused by ADEs. For patients with ADEs, the average societal cost of illness was USD $6,235.00 [5,442.8 to 7,027.2], of which direct costs were USD $2,830.1 [2,260.7 to 3,399.4] (45%), and indirect costs USD $3,404.9 [2899.3 to 3910.4] (55%). The societal cost of illness was higher for patients with ADEs compared to other patients. ADEs caused 9.5% of all direct healthcare costs in the study population [20].”
**93-year-old patient on warfarin with unstable INR**
• Patient on concomitant torsemide.
• Messaged provider regarding torsemide/warfarin interaction.
• Patient transitioned to apixaban after months of INR not within goal–INR supratherapeutic.
**Safety**: Patient at risk of bleeding, increased fall risk and potential hemorrhage.
**Financial Implications**:
“Most hospitalization expenditures after an anticoagulant-associated ADR were attributable to nursing costs (mean $33,189 per ADR) followed by pharmacy costs (mean $7,451 per ADR). ADRs which were determined to add incremental expense were associated with significant increases in total hospitalization cost (mean $118,429 vs. $54,858, p = 0.02) as well as cost after the ADR (mean $89,733 vs. $23,680, p = 0.004) compared with ADRs in which no incremental cost was determined to be incurred [21].”

The provider interview shows that the barriers to implementation of recommended changes include (1) provider decision to continue medications based on clinical judgement and patient need; (2) patient/family/provider reluctance to institute changes (“don’t rock the boat”); (3) medical mindset of prescribing medication to address clinical complaints; (4) determining who is responsible for deprescribing when multiple specialists are involved; (5) lack of clinical time; and (6) lack of clear guidelines for deprescribing.

The NM RMG Home Care group identified the need for a pharmacist to assist with medication optimization and deprescribing by offering step by step guidance through the process. The group also believe a pharmacist would be an asset to the team if they would reach out and discuss changes directly with patients, family and caregivers. This would provide patients and caregivers with a dedicated pharmacist treatment team member allowing for immediate access to address uncertainty, understanding and apprehension.

## Discussion

The forefront goal of the program was to implement and integrate a clinical pharmacist presence in a HBPC practice. Establishing this clinical pharmacy pilot demonstrated that pharmacist integration in HBPC identified opportunities to optimize patient care and potentially reduce or avoid additional healthcare spending. Another important aspect of this study was to describe a demographic that is serviced by home-care providers. As outlined, these patients are medically vulnerable, aged and have an increased utilization of medications. As the number of medications, a patient takes increases, so does the potential risk of adverse reactions or complications. It may be the perception that pharmacists review patient’s medications each time a new medication is added to their regimen. While this is true, many outpatient pharmacists lack access to patient’s EMR. Complicating this scenario, is patients who, due to cost, need to obtain medications from multiple different pharmacies. Fragmenting and the siloing of care follows. This adds complexity when attempting to perform a medication review from the outpatient pharmacy perspective. Having a pharmacist as a sentinel of where prescribing originates allows for the most comprehensive review to occur. In this pilot, about 81% of the patients received at least one medication recommendation. Of the 175 recommendations provided, 53 (30.3%) were accepted.

Comprehensive medication review has the unique ability to help identify medication problems. In an article from Castelli et al., it was noted that pharmacist inclusion in patient-centered medical homes (PCMH) is not widespread due to a lack of knowledge of the skill set and benefits [22]. In that study, in using comprehensive medication management, pharmacists were able to identify and work with the provider to resolve various medication therapy problems. There was a 98% acceptance rate of pharmacist interventions [22].

HBPC teams within the Veteran’s Health Administration have clinical pharmacy specialists [10]. They provide comprehensive medication management services to HBPC veterans [10]. A study of 79 HBPC veterans examined medication appropriateness and the degree of recommendation acceptance [23]. The acceptance rates for primary care providers were 69% [23], in contrast with 30% in our study. A recent study in the private sector had pharmacy resident-provider pairs making home visits to 25 homebound patients [24]. However, their study focused on developing a screening tool that identifies identify HBPC patients likely to benefit from in-home pharmacist review.

In our pilot study, the HBPC provider group agreed to the pharmacist recommendation 33.3% of the time for deprescribing recommendations and 45.8% of the time on dosing recommendations. When reviewing the results, the provider group felt that additional deprescribing would have occurred if the pharmacist was readily available to provide step by step instructions how to de-escalate therapy for the treatment team. Providers also agreed that recommendations would be followed if the pharmacist would act as a liaison discussing changes with the family directly. An integrated pharmacist would be an asset allowing for patient and caregiver buy-in when there is resistance or hesitancy to make changes.

Improving recommendation acceptance can occur by the pharmacist and the providers entering into a collaborative practice agreement (CPA). Initially focusing on a select few disease states, the CPA would outline a defined protocol under which the pharmacist would function to perform medication monitoring, initiating and adjusting medication regimens. Additionally, educating HBPC patients on the services provided by a pharmacist would encourage the patient to reach out to the pharmacist directly when they have any medication related problems. Patient care services provided by pharmacists and facilitated by CPA usage can assist in improving patient outcomes and reduction in the fragmentation of care [25].

Recommendation acceptance could also be increased by implementing a weekly virtual team huddle to discuss the patients sent to the pharmacist for review. Under this practice, team dialog may help to alleviate any unanswered questions that may hinder recommendation acceptance. Healthcare huddles are known for promoting patient safety, enhancing communication and fostering trust and relationship building amongst the team [26].

Home care providers are generally the sole clinician visiting the home with no additional care team members present in the home for support. The setting in which visits occur can become overwhelming depending on the environment in the home and the number of caregivers and family members present during the visit. Having immediate access to a pharmacist to assist with speaking with the family/caregiver about changes in medication therapy can be extremely valuable. This approach could become a standard of care which home care patients will grow to expect from their providers.

Our pilot program was predicated on volunteered pharmacist time, which limited the number of patients that could be reviewed on a weekly basis. Patient chart review is extremely labor-intensive as evidenced by the total and average time required to review each case. Given the population that NM RMG Home Care currently has, there exists a divide between pharmacist clinical support and the ambulatory providers/patients who may benefit from pharmacist intervention. Without access and the integration of pharmacists into the treatment team providing this type of service, providers in the field lack expert point of care access and decision-making abilities to optimize patient’s complex medication regimens and conditions.

As evidenced from comments from our own providers, seeking input from various inpatient hospital or retail pharmacists has not been a consistent or ideal state. There needs to be provision and advocacy for pharmacists embedded in the health system that can act as liaisons and consultants to provide this type of support to the multitude of patients seeking care. As the future of reimbursement shifts away from fee for service and to pay-for-performance, a pharmacist is uniquely qualified to assist providers in providing optimal care. A remote-pharmacist model permits a larger landscape than just focusing on a single disease state or discipline. It also allows for support of multiple provider practices. Many times, embedded pharmacists are fraught with assisting with insurance coverage dilemmas, prior authorizations and other administrative tasks resulting in a decrease in the time available to provide pharmacist-specific clinical interventions. The remote-pharmacist model can serve as global resource for patient referrals or questions for complex patients and oversight for essential medication management.

While many HBPC providers state they would value the addition of a pharmacist to their team, the limiting factor frequently stated are the financial resources needed to support a pharmacist. A commonly mentioned barrier is the ability to quantify and assign a dollar value to the myriad of clinical services the pharmacist provides. Using conservative estimates, pharmacist intervention in this pilot program may have resulted in health care cost avoidance in the three patient cases described estimated to be $53,000 in direct medical costs. The estimated annual cost of drug-related morbidity and mortality resulting from non-optimized medication therapy was $528.4 billion, equivalent to 16% of total United States health care expenditures in 2016 [27]. As the population ages, there will be a growing population of patients who will require multiple medications. Older adults taking multiple medications are at increased risk of adverse events as the number of their prescribed medication increases [28].

The pharmacy profession has historically been challenged to demonstrate their value with improving patient care. How is that accomplished? For the patient who has a better outcome due to pharmacist intervention, how can one quantify how the patient’s quality of life is impacted by avoiding an emergency room visit or hospitalization, better tolerating their medications or avoiding an adverse drug reaction, improved adherence and reducing overall medical cost? To paraphrase an excerpt from the book, The *Little Prince* by Antoine de Saint-Exupéry, many of the most impactful things in the world cannot be measured, seen or touched, they are just felt [29].

This study has several limitations. A small sample size was largely due to the limited staff and time available to direct toward this pilot. One pharmacist performed all the chart reviews which limited the number of patients that could be reviewed on a weekly basis. Additionally, since only one pharmacist performed each medication review, there may be biases in the types of interventions that were recommended. Patients selected represent a single HBPC practice. This cohort resides in a geographic area where there is expanded access to healthcare, this population could have better overall health status than other HBPC patients living in a different area. A noted contributor to disparities in health is differential access to care [30]. Patients selected for pharmacist review were taking several medications and considered extremely complex. Selection bias may have pushed these patients to the pharmacist for review and may not represent the majority HBPC population. Standardization criteria may help in determining which patients are candidates for review. A home-care provider screening tool could aid to streamline the process of patient referral. We relied on estimated or potential cost-avoidance from the pharmacist medication review. While additional research is needed to investigate what that actual dollar value may truly be, there are added benefits of an integrated pharmacist team member in the management of medically complex patients.

## Conclusions

Comprehensive medication review is a critical component of the healthcare continuum. The dynamic of a home-bound patient who is being serviced at home interlaces well with a remote-pharmacist model. Many HBPC patients are medically vulnerable, due to multiple comorbidities necessitating many medications. Pharmacists have a unique skill set allowing them to provide medication support to ensure HBPC patients have optimal medication regimens. However, pharmacist integration into HBPC is lacking.

Pharmacist intervention in this pilot program may have resulted in health care cost avoidance. Aligning pharmacist services to deliver support to HBPC providers can ensure the patient is treated holistically. About 81% of patients selected by HBPC providers received medication recommendations, and 30% of these recommendations were accepted by the HBPC providers. Additional research is needed to discover ways to improve recommendation acceptance and determine if aligning pharmacists to deliver support to home-based primary care providers improve the patient experience, improve clinical outcomes and reduce healthcare expenditure.
